# Effect of Single Perineural Injection of Platelet Rich Plasma on Nerve Function in Hansen’s Disease With Truncal Neuropathy: An Interventional Study

**DOI:** 10.7759/cureus.102127

**Published:** 2026-01-23

**Authors:** Anyesha Saha, Shashank Y Kothari, Swapnil Sonune, Nirendra K Rai, Reni Benny, Anuradha D Shenoy, Prasenjit Bhadra

**Affiliations:** 1 Physical Medicine and Rehabilitation, All India Institute of Medical Sciences, Bhopal, Bhopal, IND; 2 Physical Medicine and Rehabilitation, All India Institute of Medical Sciences, Nagpur, Nagpur, IND; 3 Neurology, Apollo Sage Hospital Bhopal, Bhopal, IND; 4 Spinal injuries, Sheffield Teaching Hospitals NHS Foundation Trust, Sheffield, GBR

**Keywords:** leprosy, nerve conduction study, peroneal neuropathy, platelet rich plasma, ulnar neuropathy

## Abstract

Background

A large number of people with leprosy suffer from neuropathy and its associated complications. Platelet-rich plasma (PRP) is a simple therapy by which concentrations of natural growth factors are obtained that accelerate axonal recovery. With the intent to find an effective option for the improvement of neuropathy in Hansen’s disease, perineural injection of autologous PRP was used in this study. This study aims to investigate the potential therapeutic efficacy of perineural injection of PRP in alleviating the effects of neuropathy associated with Hansen's disease.

Methodology

A cohort of 30 patients with Hansen's disease experiencing ulnar or common peroneal neuropathy were administered a single perineural injection of autologous PRP (1 ml). Evaluations were conducted by a group of three investigators at baseline, six, and twelve weeks, encompassing two-point sensory discrimination, total sensory impairment area, nerve conduction study (NCS) parameters, Screening Activity Limitation and Safety Awareness Scale (SALSA) score, hand function, and extrinsic foot muscle power. Adverse effects were meticulously recorded.

Results

Significant improvements were observed in two-point discrimination, sensory loss area, hand dynamometry scores, and foot muscle power (p<0.05). The SALSA score exhibited a significant enhancement at the 12-week mark. Motor NCS demonstrated a substantial increase in amplitude, while sensory NCS revealed a noteworthy decrease in latency and an increase in conduction velocity at the 12-week assessment. No adverse effects were documented, aside from transient pain at the injection site for one or two days.

As the follow-up period in this study was 12 weeks, the long-term effects of peri-neural injection of PRP on Hansen’s neuropathy could not be assessed. COVID-19 also had an impact on the follow-up visits of the participants, resulting in three patients being lost to follow-up.

Conclusion

This study suggests that perineural injection of autologous PRP could be a safe and promising therapeutic option for Hansen's neuropathy. However, further research and long-term follow-up studies are imperative to establish its sustained efficacy.

## Introduction

Leprosy (Hansen’s disease), a chronic infectious disease caused by Mycobacterium leprae, remains a public health concern in India despite significant strides in healthcare [1]. With the advent of multidrug therapy (MDT), leprosy's impact has dramatically decreased worldwide. The WHO reported that India had achieved the elimination goal by the end of 2005. Nonetheless, 60% of all newly reported cases globally are from India each year [2]. Leprosy primarily affects the peripheral nerves, resulting in sensory impairment and loss of muscle function, which ultimately leads to bony deformities and loss of parts of the extremities [3]. Even with the advent of MDT, ulcerations and deformities are common in leprosy patients, affecting an individual's ability to perform daily activities. The ulnar nerve, followed by the common peroneal nerve, is most involved in leprosy [4].

Corticosteroid (prednisolone) is the only drug that has been shown to improve nerve function impairment in leprosy [5,6]. Nonetheless, the degree of improvement is directly correlated with the initial severity of nerve damage upon commencement of treatment [7,8]. Therefore, an alternative therapeutic approach is required to minimize and reverse the nerve damage.

Schwann cells (SC) play a vital role in the repair and regeneration of peripheral nerves. Their functions include facilitating the recruitment of inflammatory cells, thereby forming the Bungner band to bridge nerve stumps. Additionally, SCs promote axon regeneration by secreting diverse active substances, such as neurotrophic factors, and can mature into myelin sheaths during nerve regeneration. Platelet-rich plasma (PRP) is an endogenous active substance that contains elevated concentrations of platelets, growth factors, leukocytes, fibrin, and a range of bioactive factors, such as fibronectin, osteonectin, and vitronectin, which foster SC proliferation and migration [9]. The available literature has shown promising results of PRP in traumatic and compressive neuropathy [10-12]. However, there is a lack of objective evidence regarding the effect of PRP on nerve function improvement in leprosy cases. Therefore, this study was designed to identify a safe, alternative therapeutic option for peripheral neuropathy in leprosy using perineural infiltration of autologous PRP and to assess its effects objectively with nerve conduction studies.

## Materials and methods

The study was conducted at All India Institute of Medical Sciences, Bhopal, India. Patients with Hansen’s disease, as diagnosed by skin or nerve biopsy and acid-fast staining, with involvement of ulnar and/or common peroneal nerve, were recruited in the study.

Adults diagnosed with Hansen’s disease and undergoing multi-drug therapy (MDT) were included in the study if they had peripheral neuropathy, specifically ulnar or common peroneal neuropathy, that resulted in functional impairment.

Exclusion criteria included a diagnosis of Hansen’s disease for over three years, active inflammation or abscess near the affected nerves, and ongoing lepra reactions. Participants who had infections at the injection site, received local steroid injections within six months, took nonsteroidal anti-inflammatory drugs (NSAIDs) within a week before the intervention, or were on systemic steroids exceeding 20 mg daily, were also excluded. Additional exclusions were made for those using antiplatelet medications, having uncontrolled diabetes mellitus, or known severe cardiac, hepatic, or renal conditions. Pregnant or lactating females were not included. Blood samples were analyzed for the quantity and quality of blood cells, including platelets. Individuals with abnormal hemoglobin or blood counts, such as a platelet count below 50 x 10^3, hemoglobin levels under 10 g/dl, or mean platelet volume exceeding 12.3 femtoliter, were disqualified from the study.

The study was approved by the Institutional Ethics Committee and was registered in the Clinical Trials Registry- India (CTRI/2022/11/046962). All patients were informed about the procedure followed in the study protocol to dispel any misconceptions, and informed consent was taken.

Study design

It was a prospective interventional open-label study. Thirty patients with Hansen’s disease on or post-treatment with multidrug therapy, attending the outpatient department of Physical Medicine and Rehabilitation or Dermatology between May 2021 and September 2022, with involvement of the ulnar or common peroneal nerve, were included in the study. If a patient had both ulnar and common peroneal neuropathy, then every single nerve was taken in the study as a separate sample. 

Autologous PRP was prepared by the single spin centrifugation method using the Remi R-8C^TM^ centrifuge machine (REMI Sales & Engineering Ltd., Mumbai, India).

**Figure 1:**
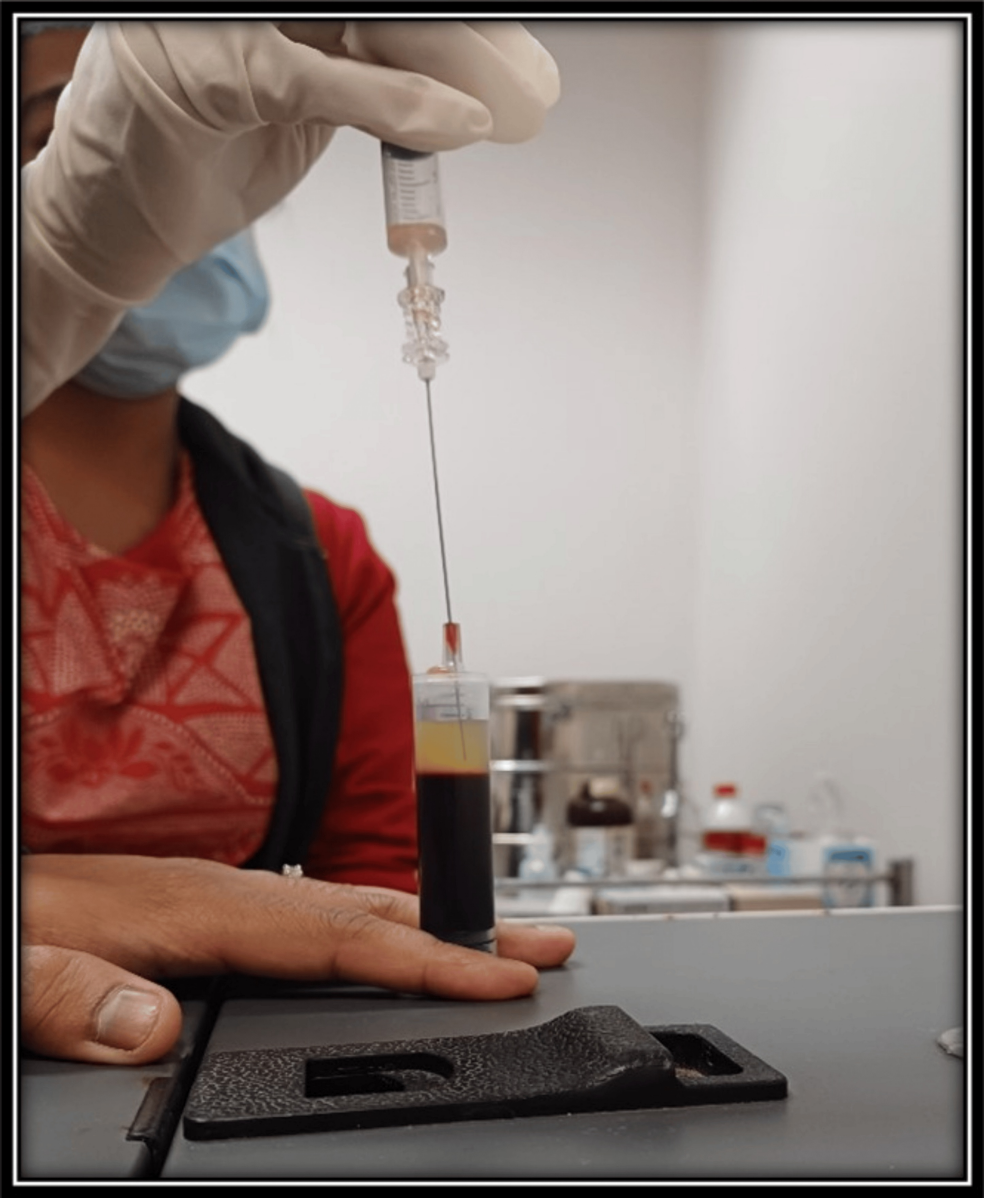
PRP aspiration from buffy coat/ RBC interface PRP: platelet-rich plasma; RBC: red blood cells.

Body-compatible anticoagulant (Citrate-phosphate-dextrose solution with adenine) was used during preparation of PRP, with patients’ blood taken in an auto-disable syringe, and the plunger was broken. The syringe was kept with the needle bent to seal the chamber, which was then placed in the centrifuge. Centrifugation was done by single spin method at 500 rpm for 15 minutes in the outswing rotor. PRP was withdrawn in a sterile syringe using a sterile lumbar puncture needle (22 Gauge) near the buffy coat/ red blood cells (RBC) interface (Figures 1).

Platelet count was done manually using microscopy, and PRP with a platelet count of less than 1,000,000 was discarded. All samples were sent for microbial culture.

One ml of freshly prepared PRP was given perineurally in the ulnar/common peroneal nerve by anatomical landmark technique within 15 minutes of preparation of PRP using a 1.5-inch 22-gauge needle. Post-injection, all the patients were observed in the department for one hour for any injection-related complications. A kit was kept handy to deal with any adverse reactions. A cold compress was given for 20 minutes over the site of injection if they had pain.

All participants in the study were instructed to limit extensive use of their hands and/or legs for the next 24 hours. Patients were permitted to continue with activities of daily living immediately. They were not allowed any non-steroidal anti-inflammatory drugs throughout the study period, but were allowed to take a Paracetamol tablet 500 mg SOS (maximum 2 g/day) as rescue medication for pain at the injection site.

All the results were recorded by a team of three investigators led by the principal investigator. The participants were questioned through semi-structured interviews. Two-Point Discrimination Test (TPDT) score, total area of sensory loss, Screening Activity Limitation and Safety Awareness Scale (SALSA) score [13], hand function using dynamometry, and foot extrinsic muscles power were assessed at the time of recruitment and reassessed at six weeks and 12 weeks after intervention. Nerve function study parameters were assessed at the time of initial assessment and at 12 weeks. The total area of decreased sensation was marked over the transparency sheet, and the area was calculated with graph paper. Two-point discrimination was measured at the area of maximum deficit within the autonomous area of the ulnar nerve and/or common peroneal nerve. The same area was noted for post-intervention comparison.

Statistics

Categorical variables were presented in numbers and percentages (%), and continuous variables were presented as mean ± standard deviation (SD) and median. The normality of data was tested by the Kolmogorov-Smirnov test. If the normality was rejected, then a non-parametric test was used. Quantitative variables were compared using the Wilcoxon signed rank test (when the data sets were normally distributed) between pre- and post-intervention, and a p-value of <0.05 was considered significant. Analysis was done using SPSS version 21.0 (IBM Corp., Armonk, New York).

## Results

**Figure 2:**
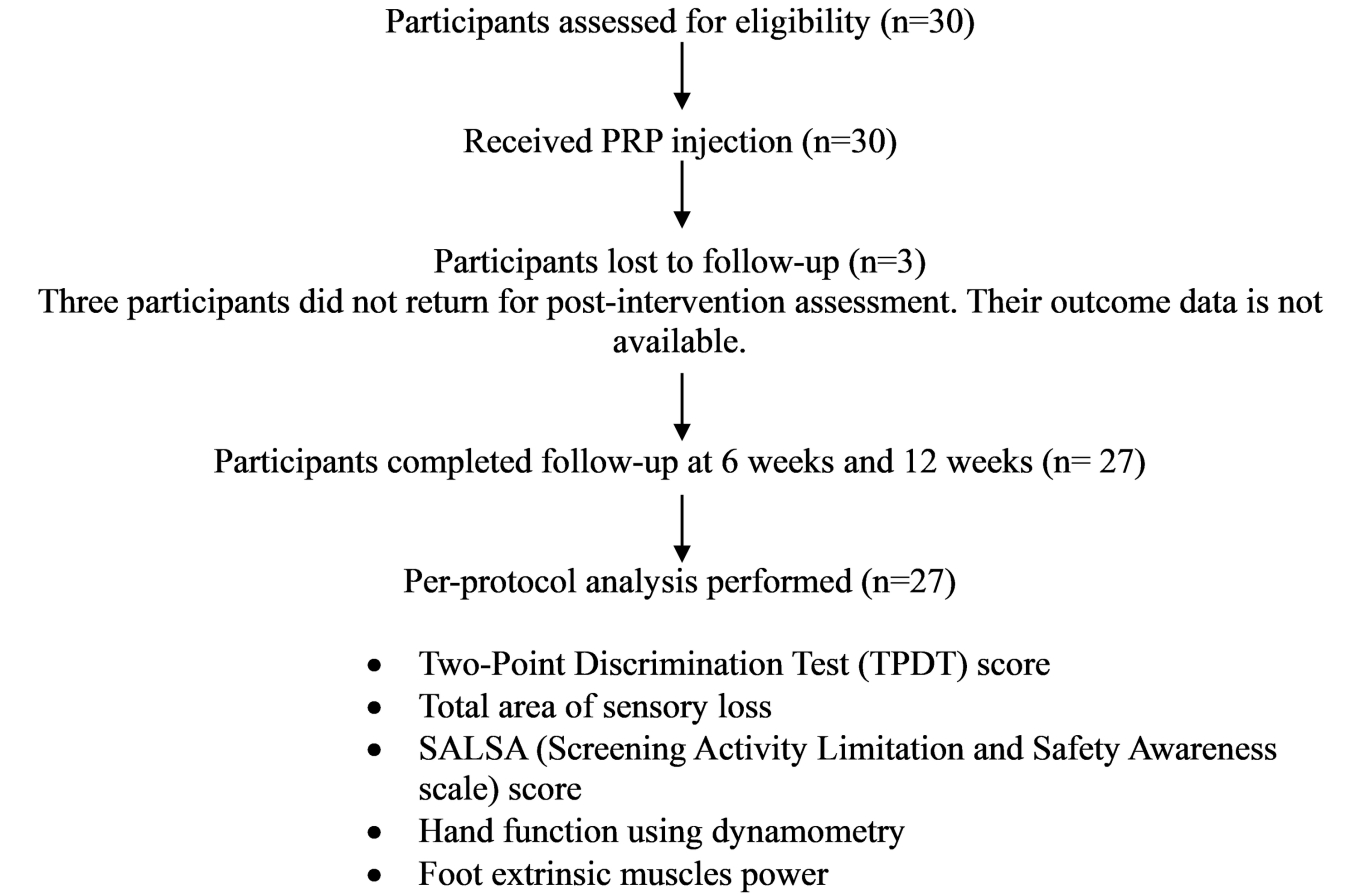
Study flow diagram

Between May 2021 and September 2022, thirty cases were recruited. Three patients were lost to follow-up (COVID-19 time); hence, a per-protocol analysis was performed on 27 patients who completed follow-up (Figure 2).

Patients included in the study ranged from 19 to 52 years of age, with a mean of 34.74 ± 12.15, and the majority of the patients were male. The right ulnar nerve was the most involved nerve, followed by the left ulnar and left common peroneal nerve. The mean duration since diagnosis of Hansen's disease was one year and 10 months (Table 1).

**Table 1:** Demographic details of participants CPN: common peroneal nerve; N=27

Demographic Characters	Frequency	Percentage (%)	Mean
Age (years)			34.74 ± 12.15
≤20	2	7.41	
21-30	12	44.44	
31-40	3	11.11	
>40	10	37.04	
Gender			
Male	24	88.89	
Female	3	11.11	
Nerve Involved			
Left CPN	5	18.52	
Left ulnar	7	25.93	
Right CPN	4	14.81	
Right ulnar	11	40.74	
Duration of diagnosis (years)			1.84 ± 0.72
<=1.5	12	44.44	
1.5-3	15	55.56	

The clinical tests, Two-Point Discrimination Test (TPDT), and area of sensory loss showed significant improvement after 12 weeks. Activity scoring using the Screening Activity Limitation and Safety Awareness (SALSA) scale showed deterioration at six weeks, but improvement was observed at 12 weeks, which was not significant (Table 2).

**Table 2:** Clinical tests and scoring TPDT: Two-Point Discrimination Test; SALSA: Screening Activity Limitation and Safety Awareness scale; SD: standard deviation. Wilcoxon signed rank test was used to calculate p-value; threshold for statistical significance: p<0.05; N=27.

Clinical test/ Week	Mean ± SD	p-value
TPDT (cm)		
0 week	4.17 ± 1.81	
6 weeks	3.86 ± 1.70	<0.0001
12 weeks	3.66 ± 1.63	<0.0001
Area of sensory loss (cm^2^)		
0 week	24.57 ± 25.36	
6 weeks	22.46 ± 23.64	<0.0001
12 weeks	21.46 ± 23.32	<0.0001
SALSA score		
0 week	35.44 ± 9.40	
6 weeks	35.93 ± 8.84	0.261
12 weeks	34.48 ± 7.81	0.051

There was improvement in finger pinch, grip strength, and the strength of the extrinsic foot muscles after 12 weeks. While the improvement in lateral pinch, three-finger pinch, and hand grip strength was significant, tip-to-tip pinch strength and power of the extrinsic foot muscles also improved at 12 weeks, though the difference was not significant (Table 3). 

**Table 3:** Comparison of strength- pinch, grip, and extrinsic foot muscles SD: standard deviation. Wilcoxon signed rank test was used to calculate p-value; threshold for statistical significance: p<0.05; N=27.

Strength variable/ Week	Mean ± SD	p-value
Pinch strength (Kg)		
Tip-to-tip pinch		
0 week	4.00 ± 2.87	
6 weeks	4.39 ± 3.31	0.038
12 weeks	5.22 ± 3.99	0.481
Lateral pinch		
0 week	4.67 ± 2.97	
6 weeks	5.28 ± 3.18	0.009
12 weeks	6.06 ± 3.21	0.001
Three-finger pinch		
0 week	3.50 ± 3.62	
6 weeks	4.22 ± 4.12	0.016
12 weeks	4.78 ± 4.01	0.002
Hand grip strength (pounds)		
0 week	19.49 ± 10.17	
6 weeks	20.43 ± 10.18	0.001
12 weeks	21.54 ± 10.01	<0.0001
Extrinsic foot muscles strength		
0 week	4.78 ± 0.44	
6 weeks	4.78 ± 0.44	1.000
12 weeks	4.85 ± 0.29	0.157

The nerve conduction study(NCS) showed significant improvement in motor and sensory amplitude and sensory Nerve Conduction Velocity (NCV). There was no significant difference in latency and motor NCV (Table 4).

**Table 4:** Nerve conduction study (motor & sensory) SD: standard deviation. Wilcoxon signed rank test was used to calculate p-value; threshold for statistical significance: p<0.05; N=27.

Nerve conduction study parameters/ Week	Mean ± SD	p-value
Nerve conduction study (motor)		
Amplitude (mV)		0.006
0 week	3.48 ± 3.61	
12 weeks	4.44 ± 4.00	
Latency (msec)		0.753
0 week	3.20 ± 2.32	
12 weeks	3.14 ± 3.02	
Conduction velocity (m/s)		0.936
0 week	25.72 ± 21.31	
12 weeks	26.83 ± 20.32	
Nerve conduction study (sensory)		
Amplitude (µV)		0.010
0 week	2.57 ± 4.69	
12 weeks	5.60 ± 10.04	
Latency (msec)		0.285
0 week	3.13 ± 1.19	
12 weeks	2.64 ± 1.27	
Conduction velocity (m/s)		0.006
0 week	17.27 ± 23.19	
12 weeks	32.63 ± 24.87	

## Discussion

Hansen's disease still affects a large number of people in India, resulting in numerous deformities and ulcers that are incapacitating. There is presently no long-term treatment for the neuropathy linked to Hansen's disease, particularly for sensory loss. The patients can benefit from any improvement in their sensory impairment because the presence of even slight sensation can stop ulcers from forming and save them from ensuing deformities. Autologous PRP is a potentially effective method for tissue regeneration. A relatively new area of interest is the effect of PRP on neural tissue. The application of PRP in neuropathies is supported by numerous animal studies. Giannessi et al. used a rat model of sciatic nerve neurotmesis-neurorrhaphy to test the use of autologous PRP suturable membrane [14]. In his opinion, PRP might improve the regeneration of peripheral nerves.

There are several approaches available for PRP preparation. We employed the slow single-spin approach in our investigation. Autologous PRP was generated using a slow single-spin process in a study by Kothari et al., and a platelet count of at least one million was typically attained [15]. We compared sensory status using a two-point discrimination test, which showed noticeable improvements at 6\six and 12 weeks in comparison to 0 weeks. Anjayani et al. conducted a similar trial in 2014, comparing the effects of 1 milliliter of PRP to a placebo in sixty patients with peripheral neuropathy due to Hansen's disease. At two weeks, there was a significant (p=0.000) improvement in the PRP group, showing that growth factors in PRP have a restorative function in neuropathy [16]. The decrease in TPDT in the area of sensory loss objectively indicates an increase in fine innervation in the area of sensory loss.

As is well known, the neuropathy linked to Hansen's disease results in ulcers, deformities, and sensory-motor deficiencies that impair one's ability to do certain activities. The SALSA scale was employed in our study to evaluate how the intervention affected the limitation of activities. The SALSA score initially showed a negligible increase at six weeks, as the patients were instructed to refrain from specific activities, such as handling hot items, due to the risk of damage in these patients. However, by 12 weeks, there was a definite increase in the score in comparison to 0 weeks, showing improvement.

In a case report, Swarnakar et al. concluded that PRP has a promising role in reducing nerve thickness and improving the sensory function in leprosy [17]. Human trials in other conditions have also shown the role of PRP in peripheral neuropathy. Sanchez et al. described a case of a young man who suffered from peroneal nerve palsy with foot drop after multiple ligament injuries to the knee. Electromyography (EMG) done after 21 weeks of intraneural injection of PRP revealed complete reinnervation for the peroneus longus and a partial reinnervation for the anterior tibialis muscle [12]. Another case report by Kuffler et al. described the use of a collagen tube filled with autologous platelet-rich fibrin to stimulate sensory and motor recovery, which healed a long segment of the ulnar nerve more than three years after the initial trauma [18]. In a clinical randomised trial, Scala et al. concluded that PRP may be protective against the neurological deficit in the facial nerve of a patient who underwent superficial parotidectomy [19]. Carpal tunnel syndrome has also been successfully treated with PRP [20]. But in this study, it was not clear if the improvement was due to nerve tissue regeneration per se or due to the anti-inflammatory effect of PRP in the tight flexor compartment. In a case report by Nashed et al., a 60-year-old leprosy patient was given an intraneural injection of steroid every month for 6 months in the ulnar and median nerves and they found significant improvement in distal motor latency and conduction velocity in the median nerve and improved compound muscle action potential (CMAP) and sensory nerve action potential (SNAP) in both ulnar and median nerves [5]. As only one such case was observed, and it involved multiple injections, it is difficult to compare its efficacy with our study. In contrast to these studies, we have treated a series of 27 cases (individual nerves) and objectively evaluated them with NCS studies, showing improvement in nerve function conclusively, in addition to clinical parameters. Our patients showed clear improvement in sensory parameters on NCS findings at 12 weeks of follow-up.

Motor assessment in the upper limb with hand dynamometry showed a significant improvement in pinch and grip strength at 12 weeks in our study, but the improvement in motor deficit in the lower limbs was not significant at 12 weeks.

We did not encounter any serious complications except mild pain at the injection site for a day or two, which needed minimal analgesics, proving the safety of PRP injection.

Limitations

The follow-up period was only 12 weeks, and long-term effects could not be assessed. A long-term follow-up with a larger study and a control group is required to establish the efficacy of perineural autologous PRP injection in truncal neuropathy of Hansen’s disease.

## Conclusions

Perineural injection of autologous PRP can be considered a safe and promising therapeutic option for neuropathy associated with Hansen’s disease. As observed on objective criteria, perineural injection of autologous PRP can improve amplitude in NCS and can improve muscle strength as observed in dynamometry. Further research in this field with a larger sample size should be considered in the future so that the results can be extrapolated to the general population of Hansen’s disease.
